# Obesity Impairs Functional Recovery of Older Stroke Patients with Possible Sarcopenia: A Retrospective Cohort Study

**DOI:** 10.3390/jcm12113676

**Published:** 2023-05-25

**Authors:** Na Young Kim, Young-Ah Choi

**Affiliations:** 1 Department of Rehabilitation Medicine, Yongin Severance Hospital, Yonsei University College of Medicine, Yongin 16995, Republic of Korea; 2 Department of Rehabilitation Medicine, Incheon St. Mary’s Hospital, College of Medicine, The Catholic University of Korea, Seoul 06591, Republic of Korea

**Keywords:** activities of daily living, rehabilitation, sarcopenia, obesity, stroke

## Abstract

The functional prognosis of older patients with coexisting obesity and possible sarcopenia remains uncertain following acute stroke. This study aimed to determine whether coexisting obesity independently affects activities of daily living (ADL) and balance ability at discharge in older patients with possible sarcopenia admitted to a stroke rehabilitation ward. A total of 111 patients aged 65 years or older with possible sarcopenia were included, of whom 36 (32.4%) had coexisting obesity. Possible sarcopenia was diagnosed based on low handgrip strength without reduced muscle mass, while obesity was determined by body fat percentage (≥25% for men, ≥30% for women). Multivariate linear regression analysis revealed that compared to patients without obesity, patients with obesity had a higher likelihood of poorer ADL (b = −0.169; *p* = 0.02) and balance ability (b = −0.14; *p* = 0.04) performance at discharge following a 4-week period of inpatient rehabilitation. These findings suggest that obesity may be a modifiable risk factor in the rehabilitation of older patients with possible sarcopenia and should be considered in the assessment of decreased muscle strength.

## 1. Introduction

Sarcopenia is characterized by an age-related reduction in muscle quantity or quality, as well as impaired motor performance [1]. In contrast, possible sarcopenia is defined as low muscle strength regardless of physical status, as suggested by the Asian Working Group for Sarcopenia (AWGS) 2019 [2]. These conditions have been associated with negative outcomes such as physical disability, hospitalization, and mortality [1]. Sarcopenia has been identified as an independent predictor of stroke severity [3] and is a potential cause of poor functional recovery [4]. Moreover, stroke sequelae may induce or worsen preexisting sarcopenia and lead to further complications such as immobilization and malnutrition [5].

The concept of “sarcopenic obesity”, first proposed by Baumgartner, is characterized by age-related loss of muscle mass and dysfunction with concomitant preexisting excessive adiposity [6]. Although the prevalence of sarcopenic obesity may vary depending on age, gender, and race, a recent systematic review reported a prevalence of approximately 11% in older patients, a population in which it is known to be associated with a high overall morbidity rate [7]. Aging and age-related changes in body composition due to sex-specific hormonal changes are possibly critical biological factors in sarcopenic obesity [8]. In addition, environmental factors, including chronic low-grade inflammation and oxidative stress, metabolism leading to insulin resistance, impaired myocyte mechanism, malnutrition and reduced physical activity, and chronic diseases such as stroke, interact with each other to exacerbate sarcopenic obesity and ultimately lead to accelerated functional decline and increased risk of diseases and mortality [8,9,10,11,12].

Traditionally, obesity has been a risk factor for various cardiovascular diseases, including stroke [13]. However, for patients with stroke, obesity may paradoxically have positive effects on mortality and functional outcomes [14,15]; nevertheless, this relationship is an ongoing debate [16]. Various factors such as body mass index (BMI) [17], body fat percentage [18], and anthropometric parameters such as waist circumference and waist-to-hip ratio [19] have been suggested as diagnostic criteria for obesity in clinical studies.

Recently, there has been an increased focus on the relevance of muscle strength rather than muscle mass because of the emerging evidence indicating that lower muscle strength may be a stronger predictor of physical performance, including movement execution and failure, and mortality among older adults residing in community settings [20,21]. Although the association between reduced handgrip strength (HGS) of the less-affected hand and worse functional outcomes of patients with stroke has been established [22,23], the impact of obesity on the functional outcomes of patients with decreased muscle strength alone has not been fully examined. Recently, the presence of sarcopenic obesity in patients with stroke has been independently associated with poor performance of activities of daily living (ADL), whereas neither isolated obesity nor sarcopenia have this association with ADL performance [24]. Therefore, the aim of this study was to investigate whether there is a difference in functional outcomes at discharge based on the presence of excessive adiposity among older patients with possible sarcopenia who were admitted to a stroke rehabilitation ward. We also aimed to determine the independent association between obesity and in-hospital rehabilitation outcomes, including ADL performance and balance ability at discharge.

## 2. Materials and Methods

### 2.1. Study Population

This was a retrospective cohort study conducted at a tertiary hospital. Inclusion criteria encompassed patients aged ≥65 years who were newly diagnosed with cerebral infarction or intracerebral hemorrhage using magnetic resonance imaging or computed tomography. These patients were admitted to the hospital for acute stroke management between January 2020 and February 2023 and subsequently transferred to the Department of Rehabilitation Medicine. Exclusion criteria encompassed individuals unable to undergo measurement due to non-cooperation during examination or absence of bioelectrical impedance analysis (BIA) measurement records. Furthermore, those lacking initial evaluations or unable to partake in discharge evaluations due to circumstances such as premature discharge before completing the 4-week rehabilitation program were also excluded. Upon admission to the rehabilitation ward, personalized goal setting was established based on functional assessments, with subsequent implementation of comprehensive rehabilitation programs tailored to each patient’s needs. These programs consisted of 30-min physical therapy and occupational therapy sessions conducted twice daily, five days a week, throughout the four-week hospitalization in the rehabilitation ward. The conventional stroke rehabilitation program encompassed various interventions, such as joint range of motion exercises, muscle strengthening exercises, balance and gait training, fine motor training for the upper extremities, and activities of daily living training. Additionally, cognitive, speech, and swallowing therapy were incorporated as deemed necessary. Data collection was performed for patients with possible sarcopenia, which was identified by HGS testing, as well as for those who underwent a BIA to determine the appendicular skeletal muscle mass (ASM) and body fat percentage. This study was approved by the Institutional Ethics Committee of our hospital and was conducted in accordance with the Ethics Code of the World Medical Association (Declaration of Helsinki). Patient consent was waived owing to the retrospective nature of the study.

### 2.2. Definition of Possible Sarcopenia and Obesity

At the time of transfer to the neurorehabilitation unit, HGS was measured, and BIA was performed. Following 4 weeks of rehabilitation, a follow-up assessment was conducted immediately before discharge. HGS measurements were performed three times for both hands using a dynamometer (Jamar, Lafayette, IN, USA), and the highest value of the three measurements was used for the analysis. The AWGS 2019 criteria define possible sarcopenia as HGS <28 kg for men and <18 kg for women [2]. ASM and body fat mass were measured using a portable BIA device (In-Body S10; Biospace Co., Ltd., Seoul, Republic of Korea). The skeletal mass index (SMI) was calculated using the ASM measured by the BIA (SMI = ASM/height in m^2^); the cutoff values for low SMI were <7.0 kg/m^2^ for men and <5.7 kg/m^2^ for women [2]. We defined possible sarcopenia using low grip strength as a criterion—in the absence of a reduction in muscle mass—to differentiate it from sarcopenia. For obesity, cutoff values for body fat percentage were ≥30% and ≥35% for men and women, respectively [18].

### 2.3. Functional Outcomes

Functional outcome measures included the Korean version of the modified Barthel index (MBI) score for evaluating ADL and the Berg balance scale (BBS) for assessing balance and the risk of falls. The MBI utilizes three distinct 5-point rating scales to evaluate ADL motor skills, including bathing, personal hygiene, and ambulation/wheelchair, with a score range of 0–5; feeding, dressing, toilet transfer, bladder control, bowel control, and stair climbing, with a score range of 0–10; and chair/bed transfers and ambulation, with a score range of 0–15 [25]. The BBS was originally designed to quantitatively assess the balance and risk of falls in older adults. It is also the most commonly used assessment tool in measuring balance ability in stroke rehabilitation. The BBS consists of a 14-item scale, and a global score is computed out of a possible 56 points [26].

### 2.4. Covariates

The following demographic and clinical variables were recorded: age, sex, stroke subtype categorized as either ischemic or hemorrhagic, stroke onset date, stroke severity as measured using the National Institutes of Health stroke scale, premorbid functional status as assessed using the modified Rankin scale, comorbidities as measured using the Charlson comorbidity index, and BMI. Motor function recovery was assessed using the Fugl–Meyer assessment. Cognitive function was evaluated using the Korean Mini-Mental State Examination, and depressive symptoms were evaluated using the Beck depression inventory (BDI). To assess the nutrition-related risk of older patients, the geriatric nutritional risk index was calculated using the following formula: 1.487 × serum albumin level (g/L) + 41.7 × present body weight (kg)/ideal body weight (height^2^ [m^2^] × 22) (kg) [27]. Additionally, swallowing function was documented based on feeding tube dependency.

### 2.5. Statistical Analysis

Data of normally distributed variables are presented as mean ± SD. Non-normally distributed data are expressed as medians and interquartile ranges. To investigate potential differences in clinical variables between patients with and without obesity, the following statistical tests are performed: Student’s *t*-test or Mann–Whitney U test for continuous variables and Pearson’s chi-square test or Fisher’s exact test for categorical variables. A multivariate linear regression analysis is conducted to determine whether obesity is independently associated with functional outcomes at discharge after adjusting for potential confounding factors, including age, sex, National Institutes of Health Stroke Scale score, premorbid disability, initial functional status, cognitive function, and depression. To assess multicollinearity among the variables in each model, we use the variance inflation factor. R software (version 4.1.2) is used for all statistical analyses. Statistical significance is set at *p* < 0.05 (two-tailed).

## 3. Results

**Figure 1:**
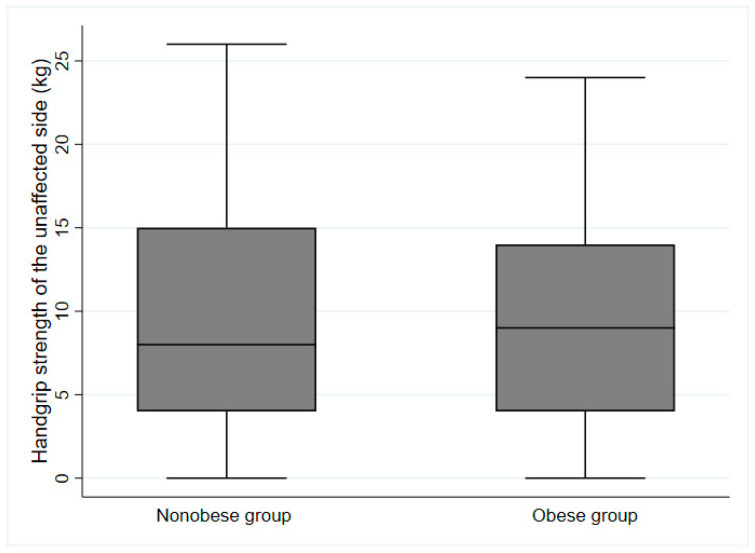
Handgrip strength of the unaffected side at baseline assessment based on obesity status (*p* = 0.371).

During the study period, 298 patients were transferred to the rehabilitation units. Ultimately, 111 patients aged ≥65 years who had possible sarcopenia were included in the analysis. Patient characteristics are summarized in Table 1 according to the presence or absence of obesity with possible sarcopenia. The proportion of patients with obesity was 32.4% (*n* = 36). The mean ages of the non-obese and obese groups were 76.9 ± 7.1 and 77.3 ± 6.0 years, respectively. Male participants comprised 37.3% (*n* = 28) and 36.1% (*n* = 13) of the non-obese and obese groups, respectively. No differences in demographic characteristics were found between the obese and non-obese groups; however, the proportion of premorbid disabilities was higher in patients with obesity than in patients without obesity (*p* = 0.031). The median BMI was 23.3 [20.2–25.0] kg/m^2^ for the non-obese group and 24.7 [22.8–27.2] kg/m^2^ for the obese group. Although there was a statistically significant difference between the two groups (*p* = 0.001), the median BMI values did not show a marked difference. Regarding the BIA measurements, the difference in mean fat mass between the two groups was larger than the difference in median BMI. The mean fat mass was 20 ± 10% for the non-obese group and 40 ± 10% for the obese group (*p* < 0.001). The height-adjusted ASM was higher in the non-obese group than in the obese group (*p* = 0.006). There were no significant differences between groups in the initial functional scores. At the baseline assessment, the median value of HGS on the unaffected side in the non-obese group was 8.0 [4.0–15.0] kg, which was not significantly different from that of the obese group (9.0 [4.0–14.0] kg; Figure 1). However, the BDI score was higher in the non-obese group than in the obese group (*p* = 0.02).

**Figure 2:**
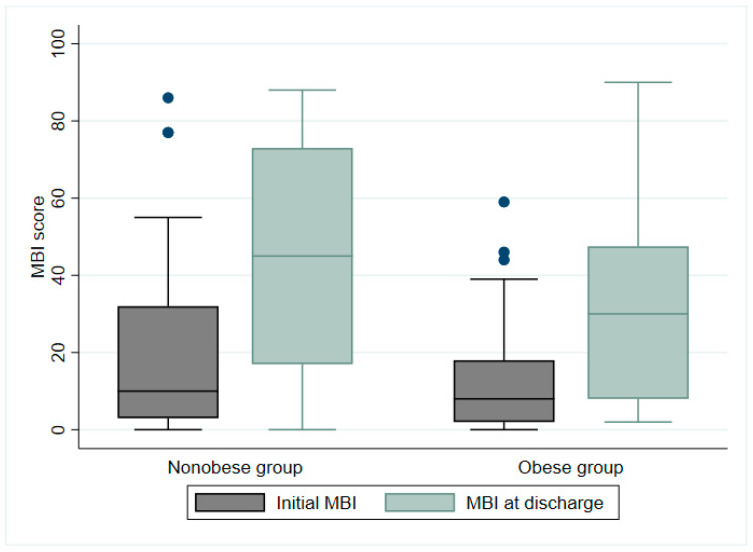
Modified Barthel index (MBI) scores of nonobese and obese groups at time of discharge following 4 weeks of inpatient rehabilitation (*p* = 0.006). Blue dots represent outliers.

**Figure 3:**
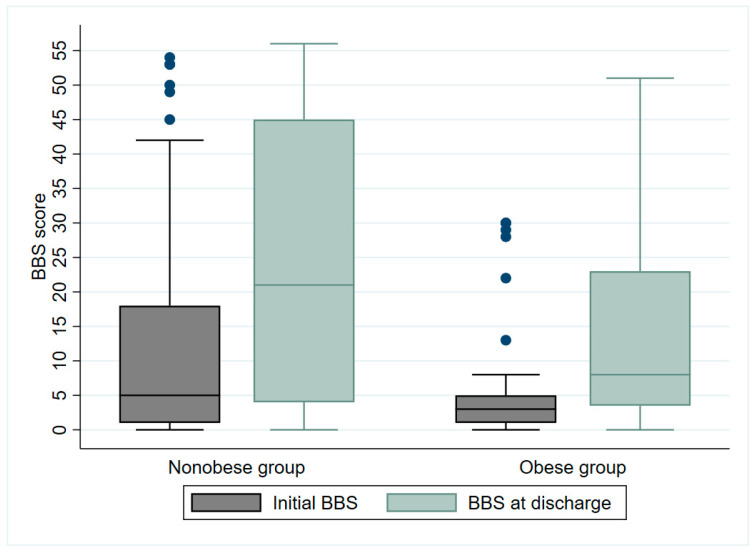
Berg balance scale (BBS) of nonobese and obese groups at discharge after 4 weeks of inpatient rehabilitation (*p* = 0.0437). Blue dots represent outliers.

At the time of discharge, the median MBI score improved from 10 to 45 points for the non-obese group and from 8 to 30 points for the obese group (Figure 2). The median BBS score improved from 5 to 21 points for the non-obese group and from 3 to 8 points for the obese group (Figure 3). Although there were no significant differences in ADL performance or balance ability between groups at the time of the initial evaluation, there were significant differences in both MBI and BBS scores at discharge, depending on the presence or absence of obesity (non-obese group: MBI: *p* = 0.006; BBS score: *p* = 0.04) after approximately 4 weeks of inpatient rehabilitation.

The results of the multivariate linear regression analysis of the MBI and BBS scores at the time of discharge are presented in Table 2 and Table 3. The evaluation of the MBI at discharge revealed that older patients (b = −0.196; *p* = 0.006) with worse stroke severity (b = −0.353; *p* < 0.001), premorbid disability before stroke onset (b = −0.184; *p* = 0.008), and obesity (b = −0.169; *p* = 0.021) had a higher likelihood of exhibiting worse ADL performance. In contrast, better initial ADL ability (b = 0.198; *p* = 0.022) and better cognitive function (b = 0.176; *p* = 0.028) were positively associated with better ADL performance at the time of discharge. Regarding balance ability assessed at discharge, the study findings showed that patients with worse stroke severity (b = −0.231, *p* = 0.003), premorbid disability (b = −0.181; *p* = 0.005), obesity (b = −0.14; *p* = 0.037), and more severe depressive symptoms (b = −0.183; *p* = 0.005) had worse balance ability at discharge, whereas those with higher initial BBS scores (b = 0.519; *p* < 0.001) had better balance ability. There was no evidence of multicollinearity in any multivariate linear regression model.

## 4. Discussion

Our study found that, among older patients with possible sarcopenia hospitalized for acute stroke, patients with obesity had worse in-hospital rehabilitation outcomes than patients without obesity. Furthermore, we demonstrated that excessive adiposity has an independent negative association with ADL performance and balance at discharge, particularly among older individuals with possible sarcopenia. Because the functional prognosis may be worse among older individuals with both reduced HGS and obesity, it is important to allocate increased attention to these patients during early rehabilitation and continually assess their progression.

A comparison of the functional outcomes at discharge among patients with possible sarcopenia showed a marked difference in ADL performance and balance ability between patients with and without obesity; however, there was no significant difference in the initial functional evaluations between groups. This result is consistent with those of a study of the general older population, which showed that the combination of low HGS and abdominal obesity is associated not only with mortality but also with ADL disability and an increased risk of falls attributable to balance control deficits. The ELSA Cohort Study in Brazil showed that the presence of both reduced HGS and excess abdominal fat was associated with a higher rate of increased ADL disability among English older adults [28]. Furthermore, in the Chinese population, older individuals with coexisting sarcopenia and obesity were at greater risk for ADL/instrumental ADL disability than those with low HGS or obesity [29]. Furthermore, in accordance with the present results, a recent cross-sectional study revealed that the risk of falls was higher among older individuals with both low HGS and abdominal obesity than among those with either low HGS or abdominal obesity alone [30].

Our study identified multiple prognostic factors related to the ADL capability at discharge during the subacute phase of stroke among older patients with possible sarcopenia. Older age, premorbid and initial ADL disability, stroke severity, and cognitive function had remarkable effects on ADL performance at discharge. A systematic review that investigated the prognostic factors affecting ambulation and ADL performance of patients with stroke during the subacute phase showed that advanced age, initial disability performing ADL and ambulating, severe paresis or paralysis, and visuospatial cognitive impairment were prognostic factors, similar to our results. The most notable outcome was that obesity with possible sarcopenia had an independent negative impact on ADL performance. For individuals with sarcopenia, obesity may exacerbate the degradation of muscle quality resulting from the infiltration of intermuscular and intramuscular fat. This process can result in dysfunction of the peripheral nerve and muscle systems, which are essential for mobility, ultimately resulting in the development of disabilities affecting the performance of ADL [31,32].

Furthermore, the inability to maintain a seated position during the early stages after stroke is a strong indicator of a negative prognosis for regaining independence in terms of mobility and ADL performance [33]. Therefore, we investigated the factors affecting the recovery of balance among patients with reduced HGS. Previous studies reported that balance disability is associated with strength, lower limb control, and ankle proprioception [34]. We found that worse stroke severity, presence of preexisting disability, and initially impaired balance all had negative effects on balance recovery, possibly because of the impairment of motor and sensory functions necessary for maintaining balance. Additionally, we identified that depression was closely associated with balance outcomes, consistent with the findings of another study [35]. Poststroke depression may affect the motivation for rehabilitation therapy and may be closely related to poor balance recovery. Furthermore, obesity is extensively correlated with balance. Obesity itself has a negative impact on balance control because excessive pressure caused by carrying excess weight may lead to diminished sensitivity in the soles of the feet and greater gravitational torque, both of which negatively affect balance control [36,37].

Although muscle mass, strength, and physical function are considered when defining sarcopenia, recent research has emphasized the importance of muscle strength in various health outcomes. Moreover, decreased lean mass is not a reliable predictor of mortality. Muscle strength, which is a marker of muscle quality, is a better indicator of mortality risk than muscle size [38]. For stroke patients, HGS on the unaffected side is linked not only to functional outcomes during hospitalization but also to poor long-term functional outcomes [4]. Furthermore, a study that examined long-term changes in HGS found that little variation occurred over time [39], indicating that HGS measured in the rehabilitation unit could have significant prognostic value.

Although the median BMI difference between the obese and non-obese groups was statistically significant (*p* < 0.05), the difference was not substantial. Instead, there was a marked difference between the two groups in body fat percentage measured using BIA. This indicates that BMI cannot adequately distinguish between fat mass and lean mass in older adults. Although BMI reflects adiposity, its sensitivity decreases particularly in older adults [40]. Recent studies recommend body composition analysis rather than BMI measurement for classifying obesity [41]. Additionally, the correlation between BMI and body fat percentage varies among different ethnic groups [42]. BMI ranges are relatively limited in Koreans, and it is known that body fat percentage has a relatively weaker correlation with BMI [43]. Therefore, in this study, body fat percentage measured by BIA was used to define obesity.

The prevalence of sarcopenic obesity is increasing in the aging population [7], and there is increasing evidence that it is associated with worse functional recovery, as shown in this study. A more detailed understanding of the complex etiology for sarcopenic obesity would enable further research into preventive strategies and treatment options. While there is currently no established treatment specifically designed for sarcopenic obesity in the elderly population, interventions targeting sarcopenic obesity focus on inducing favorable changes in body composition, including reducing fat mass and improving muscle quantity and quality. This is achieved through a combination of suitable rehabilitation exercises and nutritional interventions [8,44]. Exercise is widely recognized as the most effective approach to address sarcopenia in older adults. Engaging in appropriate aerobic exercises has been shown to enhance cardiovascular function while resistance exercises are beneficial for improving muscle strength [45]. However, due to the variability in exercise tolerance among the older population, an individualized exercise program tailored to the degree of impairment caused by stroke and the individual’s comorbidities should be provided for older stroke patients. Moreover, these patients with sarcopenic obesity are prone to malnutrition, potentially due to impaired consciousness or oropharyngeal dysphagia. Therefore, it is necessary to provide a diet with an adequate calorie and high-protein content to meet their nutritional needs [46]. The following are the clinical implications and future directions. A commonly proposed explanation for the obesity paradox is that a higher BMI suggests better nutritional status or higher energy reserves [47]. However, other studies reported that obesity is not helpful for the recovery of ADL and has a negative impact [48]; therefore, the role of obesity in the functional prognosis of stroke remains controversial. Recent studies have reported that sarcopenic obesity negatively affects ADL capabilities and dysphagia [24]. Our study demonstrated that, similar to previous studies of patients with sarcopenic obesity, functional outcomes were worse for obese patients with reduced HGS, even if they had normal skeletal muscle mass. These findings indicate that obesity should be assessed as a contextual factor that considers the presence of decreased muscle strength. We speculated that previous studies that examined the relationship between obesity and rehabilitation outcomes have been inconclusive because of the lack of consideration of skeletal muscle performance. Further investigations are required to explore the pathophysiological mechanisms underlying the effects of obesity on muscle performance.

This study had some limitations. First, the small sample size and single-center design may have reduced the statistical power of the study. However, even after fully adjusting for all possible confounders, obesity was independently associated with ADL performance and balance ability at discharge among older adults with possible sarcopenia. Second, because this was a retrospective cohort study, a risk of selection or recall bias cannot be out ruled. Third, we used the cutoff value of HGS, which is commonly used as the diagnostic criteria for sarcopenia among the general older population; however, additional research is required to determine whether the cutoff values of HGS are of comparable clinical significance to the older stroke population as to the general older population.

## 5. Conclusions

Older patients with obesity with possible sarcopenia who are hospitalized for acute stroke had poorer in-hospital rehabilitation outcomes than non-obese older patients. Furthermore, excessive adiposity was independently and negatively associated with ADL performance and balance ability at discharge, particularly among older individuals with reduced muscle strength. Therefore, careful attention should be paid to older patients with obesity and possible sarcopenia.

## Figures and Tables

**Table 1:** Baseline characteristics of older patients with possible sarcopenia.

	Non-Obese Group (N = 75)	Obese Group (N = 36)	*p*-Value
Age, y, mean (SD)	76.9 ± 7.1	77.3 ± 6.0	0.773
Sex, *n* (%)			1
Male	28 (37.3%)	13 (36.1%)	
Female	47 (62.7%)	23 (63.9%)	
Etiology, *n* (%)			0.768
Ischemic	57 (76.0%)	29 (80.6%)	
Hemorrhagic	18 (24.0%)	7 (19.4%)	
Time from onset, d, median [IQR]	14.0 [8.5–19.0]	14.0 [12.5–16.0]	0.952
NIHSS, score	6.0 [3.0–11.0]	7.0 [3.0–10.5]	0.975
Premorbid disability, *n* (%)			0.031
No (mRS score < 2)	69 (92.0%)	27 (75.0%)	
Yes (mRS score ≥ 2)	6 (8.0%)	9 (25.0%)	
CCI, score	4.0 [3.0–5.0]	4.5 [3.0–5.0]	0.261
BMI, kg/m^2^, median [IQR]	23.3 [20.2–25.0]	24.7 [22.8–27.2]	0.001
FAT%, mean (SD)	20 ± 10	40 ± 10	<0.001
SMI, kg/m^2^, median [IQR]	8.8 [7.9–9.7]	7.9 [7.2–8.8]	0.006
HGS of the unaffected side, kg, median [IQR]	8.0 [4.0–15.0]	9.0 [4.0–14.0]	0.371
FMA-UE, score, median [IQR]	19.0 [5.0–45.0]	30.0 [5.0–42.0]	0.525
FMA-LE, score, median [IQR]	13.0 [5.0–25.0]	18.0 [5.0–25.0]	0.924
BBS, score, median [IQR]	5.0 [1.0–17.5]	3.0 [1.0–5.0]	0.302
MBI, score, median [IQR]	10.0 [3.0–32.0]	8.0 [2.0–18.0]	0.204
MMSE, score, median [IQR]	13.0 [7.5–19.5]	12.5 [7.0–19.0]	0.767
BDI, score, median [IQR]	16.0 [7.0–27.5]	9.0 [5.5–16.0]	0.015
GNRI, score, median [IQR]	120.5 [115.4–124.4]	122.5 [115.4–128.7]	0.167
Dysphagia, *n* (%)			0.286
Oral feeding	51 (68.0%)	20 (55.6%)	
Tube dependent	24 (32.0%)	16 (44.4%)	

Data are expressed as percentage, mean, and standard deviation (SD), and median and interquartile range (IQR). BBS, Berg balance scale; BMI, body mass index; BDI, Beck depression inventory; CCI, Charlson comorbidity index; FAT%, body fat percentage; FMA-LE, Fugl–Meyer assessment for lower extremities; FMA-UE, Fugl–Meyer assessment for upper extremities; GNRI, geriatric nutritional risk index; HGS, handgrip strength; MBI, modified Barthel index; MMSE, Mini-Mental State Examination; mRS, modified Rankin scale; NIHSS, National Institutes of Health Stroke Scale; SMI, skeletal muscle index.

**Table 2:** Multivariate linear regression analysis of MBI at discharge.

Dependent Variables	Predictors	Standardized Coefficient b	t	*p*-Value	VIF
MBI at discharge	Age, y	−0.196	−2.82	0.006	1.14
	Female	−0.031	−0.37	0.714	1.72
	NIHSS, score	−0.353	−4.54	<0.001	1.43
	Presence of premorbid disability	−0.184	−2.69	0.008	1.1
	Presence of obesity	−0.169	−2.34	0.021	1.24
	Initial MBI, score	0.198	2.33	0.022	1.7
	MMSE, score	0.176	2.23	0.028	1.47
	GNRI, score, median [IQR]	−0.115	−1.35	0.181	1.73
	BDI, score, median [IQR]	−0.058	−0.84	0.405	1.12
	(constant)		4.65	<0.001	

BDI, Beck depression inventory; GNRI, geriatric nutritional risk index; IQR, interquartile range; MBI, modified Barthel index; MMSE, Mini-Mental State Examination; NIHSS, National Institutes of Health stroke scale; VIF, variance inflation factor. R^2^ = 0.572; adjusted R^2^ = 0.538.

**Table 3:** Multivariate linear regression analysis of BBS scores at discharge.

	Predictors	Standardized Coefficient b	t	*p*-Value	VIF
BBS scores at discharge	Age, y	−0.112	1.73	0.086	1.14
	Female	−0.033	0.42	0.674	1.71
	NIHSS, score	−0.231	3.04	0.003	1.59
	Presence of premorbid disability	−0.181	2.84	0.005	1.12
	Presence of obesity	−0.14	2.11	0.037	1.21
	Initial BBS, score	0.519	6.89	<0.001	1.21
	MMSE, score	−0.019	0.28	0.782	1.59
	GNRI, score, median [IQR]	−0.023	0.29	0.772	1.12
	BDI, score, median [IQR]	−0.183	2.84	0.005	1.14
	(constant)		2.7	0.008	

BBS, Berg balance scale; BDI, Beck depression inventory; GNRI, geriatric nutritional risk index; IQR, interquartile range; MMSE, Mini-Mental State Examination; NIHSS, National Institutes of Health stroke scale; VIF, variance inflation factor. R^2^ = 0.633; adjusted R^2^ = 0.600.

## Data Availability

All data analyzed during this study are available from the corresponding author upon reasonable request due to privacy restrictions.
